# Simultaneously double infection of oncolytic viruses leads to addition of cell death induction in glioblastoma cell lines

**DOI:** 10.1186/2194-7791-2-S1-A28

**Published:** 2015-07-01

**Authors:** Michael Ehrhardt, Julia Frank, Sigrun Smola, Norbert Graf

**Affiliations:** Department for pediatric oncology and hematology, University of Saarland School of Medicine, 66421 Homburg, Germany; Department of Virology, University of Saarland School of Medicine, 66421 Homburg, Germany

## Meeting abstract

Malignant glioblastomas are highly aggressive tumors virtually resistant to available treatments and predominantly leading to death. The standard treatment is surgical resection followed by chemo-irradiation therapy. However, the tumor always gains resistance after initial response. An improvement of outcome seems only be possible by evaluating new and innovative therapeutic strategies. An approach that has gained much interest for cancer treatment in the last years is virotherapy with oncolytic viruses. They represent a new class of anticancer therapeutics that have the ability to kill different forms of tumor cells. For three of such viruses (Reovirus, RV; Parvovirus-H, PV and Newcastle-Disease-Virus, NDV) a strong tumorlytic effect on glioma cells is described in vitro and in animal models [1]. It is known that the viruses can induce different kinds of cell death, like apoptosis or necroptosis, via different pathways [2–4].

To investigate the oncolytic capacity and the induction of cell death of RV, PV and NDV the glioblastoma cell line U87 was simultaneously infected with two viruses followed by analysis of cell survival by MTT-assay and the type of cell death by FACS staining for Annexin V and Propidium iodide. Moreover the cells were stained for virus proteins to search for double infection.

We could show that in simultaneously double infected cells, while the oncolytic effect of the stronger virus will be dominant, both viruses do induce the virus specific cell death. These effects are completely complementing each other. Furthermore we could show that the viruses can replicate at the same time in the cells.

Therefore we conclude that the use of two or more oncolytic viruses in glioblastoma therapy is a promising tool to add to the traditional therapy.
